# Metabolic Rate and Egg Production in Japanese Quails Can Be Predicted by Assessing Growth Parameters of Laying Hens

**DOI:** 10.3390/ani14020258

**Published:** 2024-01-14

**Authors:** Valeriy G. Narushin, Natalia A. Volkova, Anastasia N. Vetokh, Alan Yu. Dzhagaev, Ludmila A. Volkova, Darren K. Griffin, Michael N. Romanov, Natalia A. Zinovieva

**Affiliations:** 1 Research Institute for Environment Treatment, 69035 Zaporizhya, Ukraine; val@vitamarket.com.ua; 2 Vita-Market Ltd., 69035 Zaporizhya, Ukraine; 3 L. K. Ernst Federal Research Centre for Animal Husbandry, Dubrovitsy, Podolsk 142132, Moscow Oblast, Russia; anastezuya@mail.ru (A.N.V.); alan_dz@inbox.ru (A.Y.D.); ludavolkova@inbox.ru (L.A.V.); n_zinovieva@mail.ru (N.A.Z.); 4 School of Biosciences, University of Kent, Canterbury CT2 7NZ, UK; d.k.griffin@kent.ac.uk

**Keywords:** Japanese quail, *Coturnix japonica*, egg productivity, morphometric parameters, body weight, body volume, body surface area, metabolic rate, metabolic index

## Abstract

**Simple Summary:**

Quails are becoming increasingly popular for their meat and eggs, and thus, the productivity of laying hens, and how that can be predicted, is of growing interest to quail producers. Because of this, we wanted to find out whether we could predict the performance of laying hens (typically expressed as the number of eggs produced multiplied by the egg weight—the so-called total egg mass) simply by looking at certain growth traits (i.e., body weight, surface area, and volume), as well as the metabolic rate among eight Japanese quail breeds. To succeed in this analysis, we developed a novel method for calculating the volume and surface area of a quail body. As a result, we derived a new mathematical formula called the metabolic index, which included the measurements of body weight, surface area, and volume. We discovered that the total egg mass in quails can be judged from these growth parameters, particularly when we examined the slope angles of the trend lines in the graphs pertaining to these parameters.

**Abstract:**

The aim of the current study was to assess the female metabolic rate and test the hypothesis that there is a relationship between the egg productivity of Japanese quails from eight breeds and their morphometric, or growth, parameters. Parameters measured were body weight (*B*), volume (*V*), and surface area (*S*), as well as the metabolism level expressed by the ratio *S*/*V*. The collected egg performance traits were as follows: the number of eggs produced (*N*), the average egg weight (*W*), and the total egg mass (*M*) (i.e., *N* multiplied by *W*). To measure the *S* and *V* values, a novel technique was developed that takes into account the similarity of the quail’s body to an ellipsoid. An analysis of the relationships between productivity indicators allowed us to introduce a new index called the *metabolic index*, *B·S*/*V*, based on all three main growth parameters in quails. Using the values of this index, we were then able to judge indirectly the level of quails’ egg productivity. We went on to assess the *N*, *W,* and *M* values, not only depending on the size of the bird’s growth parameters but also according to the degree of their changes during quail growth. These changes were expressed as the slope angles of trend lines describing the growth process data. This approach produced more accurate results for predicting the egg productivity in terms of *W* and *M*.

## 1. Introduction

Among poultry species, Japanese quails (*Coturnix japonica* Temminck & Schlegel, 1848) have the lowest body weight (*B*). This small size implies a much larger metabolic load when laying eggs compared to larger species such as chickens. Indeed, the physiological process of egg formation requires considerable energy, nutrients, and resources fueled by reserves built up in the body prior to the onset of egg development [1]. While the consumption of nutrients necessary for egg production (*N*) in a commercial quail flock can be regulated by specialized feeding programs, the quails’ energy resource is a function of their body parameters.

Analyzing the relationship between egg weight (*W*) and *B* of a female and based on the data from a large sample of wild bird species, Rahn et al. [2] obtained an allometric equation reflecting the relationship of the percentage egg weight (*W*%) vs. *B* as follows:

(1) $$W\%=27.7B^{-0.23}.$$

An analysis of Equation (1), in particular, the negative exponent at *B* of birds, indicates that the lower the *B* of a hen, the larger the egg (in percentage terms) she produces.

According to many studies, the energy expenditure of birds, including that required for *N*, can be estimated based on the metabolic rate, *H* [3,4,5], i.e., the higher the metabolic level of a mother, the higher her energy potential. Accordingly, the females of poultry species can expend more energy on laying eggs under industrial conditions. Furthermore, it is evident that the metabolic body weight (equal to *B*^0.75^) and the total egg mass (*M*) has a stronger influence on the expected feed intake than does body weight gain (Δ*B*) as was shown in two contrasting varieties of Japanese quails [6].

Rahn et al. [7] determined that the *H* value can be calculated based on the *B* of a female using the following allometric dependence:

(2) $$H=3.5B^{0.71}.$$

It was thence demonstrated that the *W* of an egg laid directly depends on the *H* of a hen [7] and is as follows:

(3) W=0.074H.

Despite the widespread use of allometric dependencies in the study of various aspects of biology [8], some researchers have historically been skeptical about the use of the allometric approach in determining the levels of metabolism [9,10,11].

Glazier [12] reviewed the various approaches for defining *H* in animals and identified surface area (*S*) and volume (*V*) of a body as the most important morphometric factors, limiting the ability to describe metabolic processes using conventional scaling methods. The most harmonious, accessible, and logical relationship between two such characteristics is their ratio, *S*/*V*. The total rate of body metabolism can be characterized by monitoring the rate of oxygen uptake by the lungs [13] or by the amount of heat generated [14]. Both of these processes are quite accurately characterized by the *S*/*V* value; for example, the larger *S*/*V* is, the more is the *S* per *V* unit through which oxygen diffusion [15] or the change in thermal conductivity [16] that can occur.

Approaches for determining morphometric parameters, in particular *S*, have been explored for the purposes of analyzing metabolism levels in birds. There have been a few studies dating back more than 90 years [17,18] that establish the relationship between *S* and *B* in birds using allometric dependencies. As described above, the application of the allometric method using the main birds’ parameter *B* requires some revision for analyzing *H*. In this respect, new methodological approaches are required to determine the values of *S* and *V* in hens.

With the above in mind, the aims of the current research were as follows:

(1) To test the hypothesis that there is a relationship between egg productivity and the metabolism level in Japanese quails of various breeds such that *M* can be predicted through the accurate assessment of *S* and *V* of hens’ bodies;
(2) To develop a robust theoretical and methodological basis for determining *V* and *S* in Japanese quail hens such that egg productivity can be accurately predicted.

## 2. Materials and Methods

### 2.1. Experimental Birds

Eight breeds of Japanese quail listed in Table 1 were examined. The quails were kept in the vivarium of the L. K. Ernst Federal Research Centre for Animal Husbandry (LKEFRCAH), Russia. Female quails were housed individually in multi-tiered cages that were divided into compartments measuring 22.5 × 40 cm (with an area of 900 cm^2^ per bird). The conditions for keeping laying quails were standardized for all individuals: moderate fluorescent artificial lighting for 16 hours and no more than 20 lux around the feeders, ambient temperature varied from 20 to 25 °C, and humidity from 55 to 65% with well-functioning supply ventilation. The windows in the quail premise were entirely sealed from natural light. The quails had free access to commercial complete feed containing a metabolizable energy of 2850 kcal/kg and 28% crude protein at the age of 0–3 weeks, 2800 kcal/kg and 24% crude protein at 3–6 weeks, and 2900 kcal/kg and 18% crude protein after 6 weeks of age. Free access to nipple drinkers was available at all stages of quail keeping.

### 2.2. Use of Mathematical Similarity Principles

When carrying out similar research, Mitchell [17] and Perez et al. [18] used dead individuals. This, of course, in no way, is acceptable within the framework of our study as the purpose is to predict future egg productivity. In addition, in order to avoid unnecessary stress on females, we had limited time for gathering various morphometric data, which, accordingly, affected the number of animals that we could assay. The solution was to use the following fundamental approaches:

i. Among body measurements, we focused on the bird’s body, leaving out parts such as the head, legs, and unfolded wings. This constitutes about 95% of the total bird’s *V* and contains the main internal organs responsible for metabolic processes. The commercial management of quails prevents them from actively using their wings and, consequently, hampers their effect on *H*.
ii. The calculation of the *S* and *V* values were performed according to a single mathematical principle that made it possible to level out any possible errors in their measurement.
iii. For calculations, we used the principle of mathematical similarity.

**Figure 1:**
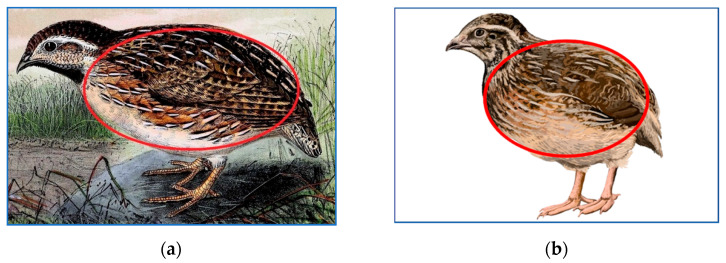
Two related *Coturnix* species, the wild common (*C. coturnix*; (**a**)) and the domestic Japanese (*C. japonica*; (**b**)) quails, with a schematic representation of the ellipse (red line) based on the measurements of the bird’s body length and chest circumference. Image sources: (**a**) https://commons.wikimedia.org/wiki/File:Coturnix_coturnix_1873.jpg (accessed on 12 January 2024), John Gerrard Keulemans (1842–1912), Onze vogels in huis en tuin (1873), Creative Commons Public Domain Mark 1.0 license (CC-PD-Mark) and (**b**) https://commons.wikimedia.org/wiki/File:202205_Japanese_quail.svg (accessed on 12 January 2024), DataBase Center for Life Science (DBCLS), https://doi.org/10.7875/togopic.2022.186 (accessed on 12 January 2024), Creative Commons Attribution 4.0 International license (CC-BY-4.0).

With the above in mind, we limited ourselves to measuring two quail morphometric parameters, i.e., body length (*l*) and chest circumference (*c*) at the widest point. An ellipsoid was chosen as the most similar mathematical figure as can be seen for both the domestic Japanese quail (*C. japonica*) and its wild relative, common quail (*C. coturnix*) in Figure 1. The *c* value was chosen as the base measurement due to the fact that the quail’s body is not perfectly round. Because of that, the ellipsoid breadth (*b*) was recalculated as the ratio *c*/*π*.

### 2.3. Determination of S and V of the Quail Body

Two basic morphometric characteristics, *S* and *V*, were calculated in accordance with the ellipsoid formulae, widely used in our previous studies (e.g., [33]), and adapted to the measured traits, in our case, *l* and *b*:

(4) $$S=\frac{\pi lb}{2}\left(\frac{\arcsin\sqrt{1-\frac{b^{2}}{l^{2}}}}{\sqrt{1-\frac{b^{2}}{l^{2}}}}+\frac{b}{l}\right),$$

(5) $$V=\frac{\pi lb^{2}}{6},$$

where *S* is the surface area of the ellipsoid (in cm^2^), *V* is the volume of the ellipsoid (in cm^3^), *l* is its length (in cm), and *b* is its maximum breadth (in cm).

A similar approach has also been recently implemented by Eichenwald and Reed [34] to determine *V* and, consequently, the bird’s density (*D*). As a result, we determined that the use of an ellipsoid for such calculations was appropriate and independently verified. Once we had developed a methodology for determining metabolic level indicators, it was possible to move on to an experimental procedure.

### 2.4. Measured Traits and Statistics

The *N* values of laying quails were assessed at the age of 3 to 5 months, i.e., as the number of eggs laid by one bird during three months of the productive period (provided in Table 2). Egg collection and individual *N* counts were performed daily. Each egg was weighed (*W*) on laboratory electronic scales. Eggs with a shelf life of no more than two days were used for evaluation. The *M* indicator for three months of observations was produced as the main criterion for quail egg productivity using the following formula: *M* = *N*·*W*.

At the age of 1 week to the start of laying (8 weeks), the weighing of quails was carried out with an interval of 1 week on laboratory electronic scales with a division value of 0.1 g. The linear measurements of *l* and *c* were produced at the age of 2, 4, 6, and 8 weeks using a measuring tape. The former (*l*) is an indicator related to the bird’s size and the development of internal organs and was assessed as the distance between the last cervical vertebra and the end of the coccyx. The latter (*c*) characterizes the development of internal organs and the strength of the physique and was determined at the base of the wings along a line passing by the last cervical vertebra and the anterior end of the keel.

The STATISTICA 5.5 software (StatSoft, Inc./TIBCO, Palo Alto, CA, USA) and Microsoft Excel computational programs were used to process the experimental data. Hereby, the validity of the obtained relationships was assessed by the value of the Pearson correlation coefficient (*R*) and regression models using the coefficient of determination (*R^2^*), with the confirmation of their significance set at the level of *p* < 0.05.

## 3. Results and Discussion

### 3.1. Changes in B, V, and S

Body measurement data for the quails of eight breeds, as well as the indicators of their productivity, are presented in Table 2.

**Figure 2:**
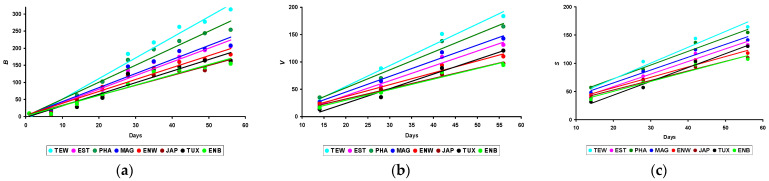
Linear changes in growth parameters among eight quail breeds: (**a**) body weight (*B*), (**b**) body volume (*V*), and (**c**) body surface area (*S*). Breeds: TEW, Texas White; EST, Estonian; PHA, Pharaoh; MAG, Manchurian Golden; ENW, English White; JAP, Japanese; TUX, Tuxedo; and ENB, English Black.

For an ease of perception of the data in Table 2, the visualization of trends in morphometric, or growth, parameters of quails (*B*, *V*, and *S*) is shown in Figure 2.

An analysis of dependencies showed that the *V* and *S* values had more pronounced differences between breeds than the *B* values. Even at the early stages of quail development, the *V* and *S* indicators differed significantly for almost all breeds.

It is interesting to note that for all considered morphometric indices of quails (*B*, *V*, and *S*), the growth lines had a different trend, i.e., slope angle. This is especially noticeable when analyzing changes in quail *B* (Figure 2a). This feature made it possible to include another group of parameters in correlation studies, i.e., the slope angle of the trend line for *B* (TAN*B*), *V* (TAN*V*), and *S* (TAN*S*), respectively.

### 3.2. The S/V Ratio and Metabolism Level

**Figure 3:**
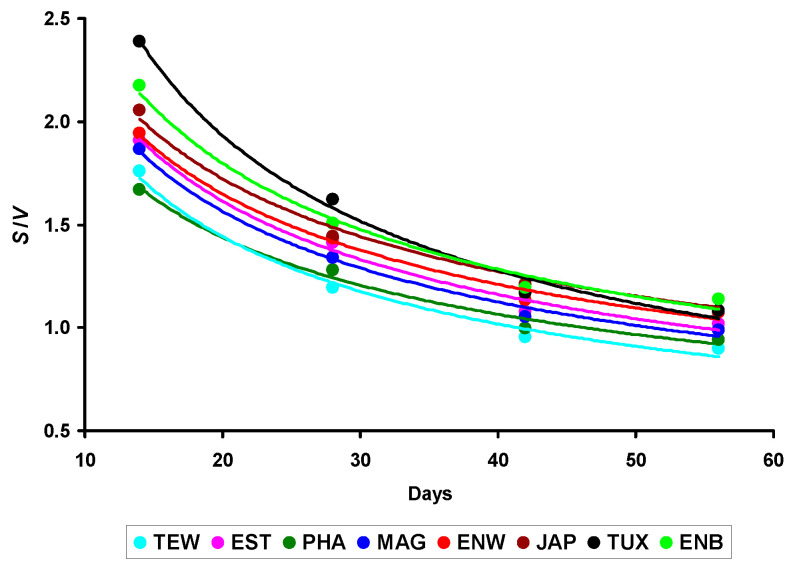
Changes in the *S*/*V* ratio among eight quail breeds. Breeds: TEW, Texas White; EST, Estonian; PHA, Pharaoh; MAG, Manchurian Golden; ENW, English White; JAP, Japanese; TUX, Tuxedo; and ENB, English Black.

When analyzing the graphical representation of trends in the *S*/*V* ratio changes, the mode of these changes turned out to be more similar for almost all breeds, especially if they were presented non-linearly, and in the form of power–law dependencies (Figure 3).

**Figure 4:**
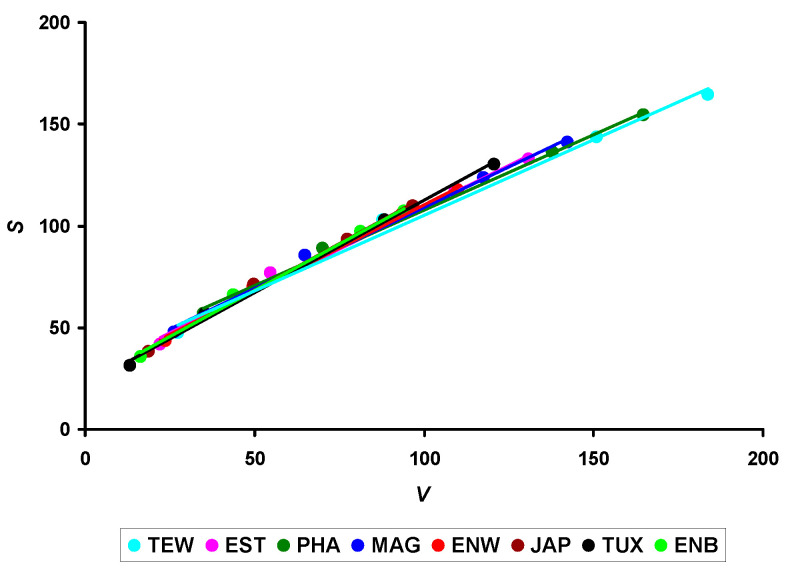
Relationship between body surface area (*S*) and volume (*V*) among eight quail breeds. Breeds: TEW, Texas White; EST, Estonian; PHA, Pharaoh; MAG, Manchurian Golden; ENW, English White; JAP, Japanese; TUX, Tuxedo; and ENB, English Black.

Obviously, the nature of the change in this indicator (*S*/*V*) and, accordingly, in the metabolism level of the birds during the entire life period can be considered more or less stable for the Japanese quail species in general. However, clear differences between breeds were noted in the *S*/*V* values. For instance, for some of the examined breeds, there was a clear dominance of the *S* value over *V*. To evaluate this process in more detail, we constructed the functional dependencies, *S* = *f*(*V*) (Figure 4).

An analysis of relationships in Figure 4 demonstrated an almost complete identity of functional changes in the *S* vs. *V* values, regardless of them belonging to different breeds. Such a coincidence of relationships between these indicators across the entire diversity of the considered sample of quail breeds allowed us to conclude that the metabolism level within this bird species is identical, despite the breed differences in growth indices and productivity level. However, it is worth acknowledging that the sample sizes of the ENB and MAG breeds were small. The representativeness of these two samples cannot be calculated, and it would be reasonable to confirm the respective breed results using larger sample sizes.

### 3.3. Correlation between Performance Traits and Morphometric Characteristics

**Figure 5:**
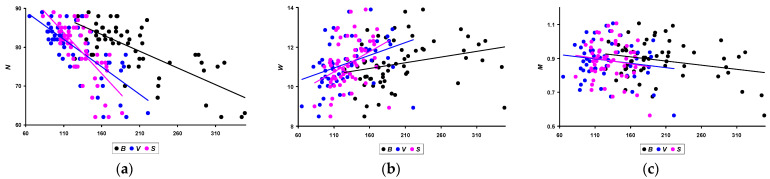
Dependencies for performance traits, i.e., (**a**) number of eggs laid (*N*); (**b**) average egg weight (*W*); and (**c**) total egg mass (*M*) for three months of the assessed period, respectively, relative to the body weight of quails (*B*), volume (*V*), and surface area of their body (*S*).

The next stage of analysis of the results obtained was to determine possible correlations between quail productivity indicators (*N*, *W*, and *M*) and other parameters measured or calculated during the process of these studies. When conducting a correlation analysis, the entire set of data obtained for the eight studied quail breeds was used. Their graphical visualization was chosen as the most suitable option for presenting the correlation dependencies. The number of eggs laid (*N*), the average *W* value, and *M* for three months of the assessed period most correlated with the *B* of quails and the *V* and *S* of their body measured on the 56th day of their life, i.e., practically at the beginning of egg laying. The corresponding graphical relationships between these indicators are presented in Figure 5.

In quantitative terms, the values of the Pearson correlation coefficient were as follows:

- *N*: −0.730 (*p* < 0.05) for *B*; −0.724 (*p* < 0.05) for *V*; and −0.708 (*p* < 0.05) for *S*.
- *W*: 0.271 (*p* < 0.05) for *B*; 0.370 (*p* < 0.05) for *V*; and 0.384 (*p* < 0.05) for *S*.
- *M*: −0.254 (*p* < 0.05) for *B*; −0.160 (insignificant) for *V*; and −0.135 (insignificant) for *S*.

Interestingly, all correlation coefficients for *N* had negative values, and accordingly, the trend lines had a downward slope. In other words, the smaller the *B* of quails and, accordingly, their *V* and *S*, the greater the number of eggs they lay. Obviously, a fairly high correlational dependence for the *N* value also influenced a similar trend in relation to *M* of all eggs laid during the three months of the productive period.

In spite of the apparent illogical nature of these findings, a similar relationship was also noted in some former studies. For example, Vieira Filho et al. [35] demonstrated that, while laying phase productivity of Japanese quails was considerably decreased when their body weight was less than 140 g at 42 days of age or less than 120 g at 35 days of age, egg production (i.e., the number of eggs laid during the laying period) in light and medium breeds was higher than that of heavy and very heavy breeds. Similar results were also obtained by Lukanov et al. [36]. Of the three heavy Japanese quail populations assessed, quails from the GL breed, which had the lowest *B* value, demonstrated the highest intensity of egg laying.

**Figure 6:**
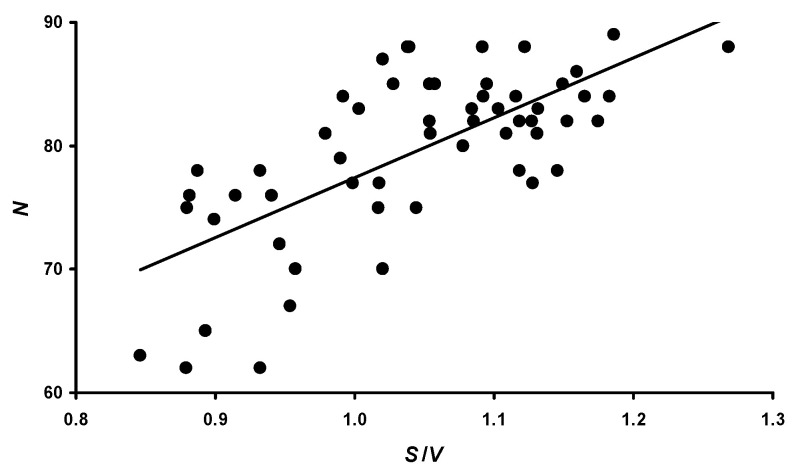
Correlation between the number of eggs laid (*N*) over three months of the assessed period and the metabolic level of quails, expressed by the *S*/*V* ratio.

We hypothesized that a possible reason for the observed inverse correlation for *N* was the metabolic rate, which we recorded as the *S*/*V* ratio. The correlation between this indicator and the *N* value is shown in the form of a graphical dependence in Figure 6.

In quantitative terms, the value of the Pearson correlation coefficient between *N* and *S*/*V* was positive and amounted to 0.696 (*p* < 0.05).

### 3.4. Inferring the Metabolic Index B·S/V

**Figure 7:**
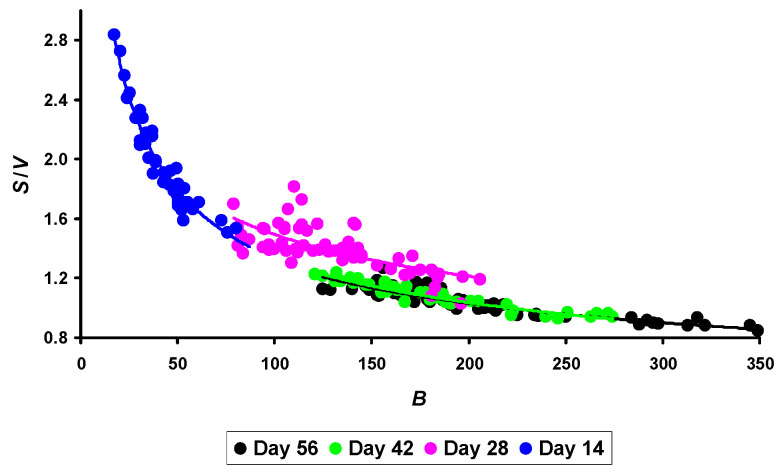
Correlation of the metabolism level of quails, expressed by the *S*/*V* ratio, with the body weight of laying hens (*B*).

The above resulting relationship is quite logical, demonstrating an increase in *N* of laying hens with an increase in their metabolic level. Obviously, it is this parameter (*S*/*V*), and not the bird’s *B* that is key when estimating the number of eggs laid. To finally answer questions about the inverse relationship between *N* and *B*, we assessed the relationship between the bird’s *B* and its metabolic level (*S*/*V*), and the visualization of which is presented in Figure 7. At the same time, we assessed the relationship between these parameters not only on the 56th day of quails’ life but also for all other measurement time points, i.e., the 14th, 28th, and 42nd days. Furthermore, taking into account that almost all studies aimed at assessing the relationship between bird’s *B* and its metabolism level established a power–law relationship between these parameters [7,12], we also provided a preference to this function.

In quantitative terms, the obtained values of the Pearson correlation coefficient are as follows: −0.899 (*p* < 0.05) for 56 days; −0.948 (*p* < 0.05) for 42 days; −0.736 (*p* < 0.05) for 28 days; and −0.916 (*p* < 0.05) for 14 days of age.

If we combine all measurements into a single numerical series, the obtained values can be approximated quite accurately by the following equation:

(6) $$\frac{S}{V}=7.224B^{-0.356},$$

where *S* is the surface area of the quail’s body (in cm^2^), *V* is the volume of the quail’s body (in cm^3^), and *B* is the body weight of quails (in g). All sizes conformed to the time interval from 14 to 56 days of the bird’s life.

The coefficient of determination (*R*^2^) for Equation (6) was 0.921 (*p* < 0.05).

Despite a fairly accurate mathematical description of the relationship between *S*/*V* and *B*, the logic of Equation (6) is open to reasonable criticism, because the exponent (−0.356) destroys the fundamental foundations of ‘a general theory of life’ (e.g., [12]), according to which the exponent should be equal to ¾ (i.e., 0.75). The foundations of this theory were laid more than 90 years ago through the work of Kleiber [37] and were later confirmed [38]. Although an increasing number of recent studies disagree with the value of ¾ [39,40], a negative value of the exponent is unacceptable in any case. For poultry, Glazier [12] provides the power–law coefficient that ranges from 0.64 to 0.66. Rahn et al. [7] provided a slightly higher value that amounts to 0.71. Obviously, the *S*/*V* ratio cannot fully serve as an indicator of the level of metabolism, at least in quails. We decided to solve the discrepancy that arose by using some mathematical transformations of Formula (6), multiplying both its parts by the *B* value. As a result, Equation (6) took the following form:

(7) $$\frac{SB}{V}=7.224B^{0.644}.$$

The exponent (0.644) in Equation (7) fully fits into the concept of the mathematical relationship between the metabolic rate (*H*) and the *B* of a bird, from which we assumed that the indicator replacing the *H* value in our case could be a precise relationship of the three main measurements of quails expressed by their ratio *B*·*S*/*V*, which we conventionally called the *metabolic index*. Undoubtedly, the purely mathematical approach that we used cannot serve as the ultimate truth as this is just a working hypothesis that requires additional experiments related to measuring the actual energy expenditure of the bird, the value of which is used to calculate the true metabolic level value. However, it should be noted that Kleiber [37], when deriving his fundamental formula, used a mathematical transformation of the surface metabolism value, after which he expressed *S* through *B* of an organism under study. In addition, taking into account all three main characteristics of the bird (*B*, *S,* and *V*) in the resulting index, we thus find a consensus among supporters for calculating the metabolic rate using *B* [7,37,38] and those who proposed to base it on the magnitude of *S* and *V* [12,13,14].

### 3.5. Correlation between the Metabolic Index B·S/V and Performance Traits

**Figure 8:**
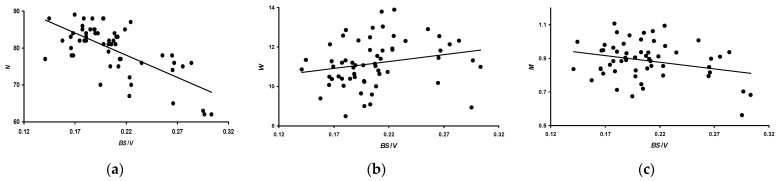
Dependencies for performance traits, i.e., (**a**) number of eggs laid (*N*); (**b**) average egg weight (*W*); and (**c**) total egg mass (*M*), for three months of the assessed period relative to the quail metabolic index *B·S*/*V*.

Based on the proposed *B·S*/*V* index, which, according to our assumption, more adequately assesses the metabolic level of quails, we analyzed its correlation with productivity indicators *N*, *W*, and *M* (Figure 8). When calculating the *B·S*/*V* index, the *B* value was taken in kg.

In quantitative terms, the values of the Pearson correlation coefficient were as follows:

- *N*: −0.690 (*p* < 0.05).
- *W*: 0.219 (insignificant).
- *M*: −0.275 (*p* < 0.05).

As observed in the analysis shown in Figure 8 and based on the nature of changes in the metabolic index, quails invest most of their reproductive energy in *W* (Figure 8b) rather than in the *N* level (Figure 8a). Considering both of these biological processes together, it can be assumed that the costs of producing higher-quality eggs are offset by a decrease in their quantity. It is possible that this feature of the body of domestic quail females comes from the physiology of wild species. For example, as reviewed by Sockman et al. [5], reproductive efforts are more aimed at warranting the egg size and, possibly, the regulation of the sex of future offspring. The number of eggs in a clutch matters only for the subsequent level of metabolism during the phase of raising offspring. After all, raising five chicks is a much more expensive investment than raising four [5]. Since domestic quail breeds are deprived of the opportunity to subsequently care for their offspring, their *N* level is most likely regulated to a greater extent by factors other than the energy costs of laying eggs.

If we distribute the component values in our proposed metabolic index in a slightly different way, it can be written in the following form:

(8) $$\frac{B}{V}\cdot S=D\cdot S,$$

where *D* is the bird’s body density in g/cm.

**Figure 9:**
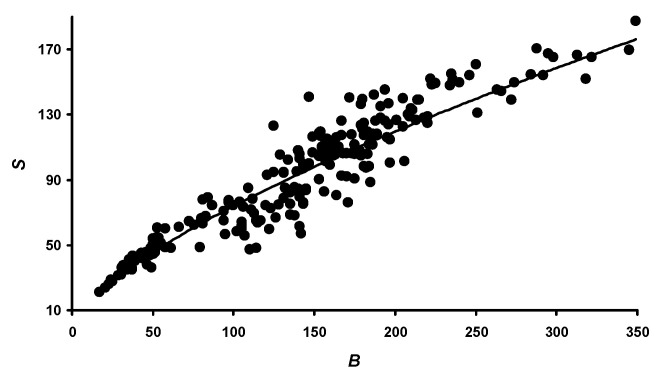
Graphical dependence of the surface area (*S*) of a quail on its body weight (*B*).

Considering that the *D* value is quite stable [41] and taking into account Equation (7), the attempts of the other authors [17,18] to link the calculation of a bird’s *S* with its *B* become understandable. We also decided to carry out a similar procedure using data from the measurements of these indicators at different periods of quail growth (Figure 9), which were approximated by the following equation:

(9) $$S=3B^{0.695},$$

where *S* is taken in cm^2^, and *B* is taken in g.

The coefficient of determination (*R*^2^) of Equation (9) was 0.906 (*p* < 0.05).

The exponent for quail’s *B* in Equation (9) is quite similar to those obtained by other researchers, i.e., 0.706 [17] and 0.559 [18]. However, the value of the coefficient in front of a bird’s *B* (in our case 3) is slightly lower. Obviously, this is explained by the fact that other authors [17,18] took into account the total *S* of the body, including the wings, which significantly increases the results of *S* measurements.

### 3.6. Examining Slope Angles for B, V, and S Changes

**Figure 10:**
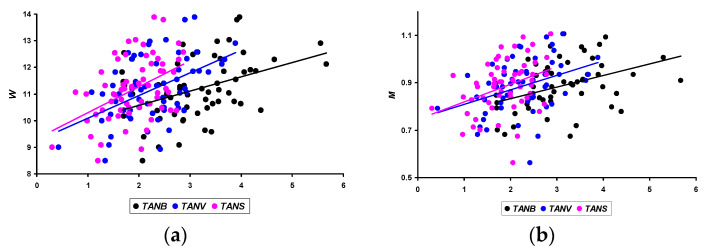
Correlation dependencies of the average egg weight, W (**a**), and the total egg mass for three months, *M* (**b**), with the slopes of trend lines reflecting changes in weight (TAN*B*), volume (TAN*V*), and surface area of quail body (TAN*S*).

When analyzing above the nature of the obtained linear dependencies for changes in growth parameters of quails (Figure 2), i.e., *B*, *V,* and *S* of the body, we noted the possible prospects of including in the analysis not only the measurement data of these indicators but also the slope angles of their trend lines: TAN*B*, TAN*V*, and TAN*S*. The calculation of these values and their correlations with the productivity parameters of quails made it possible to establish their greatest closeness with the *W* value of laid eggs and *M* for three months of the assessed period (Figure 10).

In quantitative terms, the Pearson correlation coefficient values were as follows:

- *W*: 0.399 (*p* < 0.05) for TAN*B*; 0.495 (*p* < 0.05) for TAN*V*; and 0.455 (*p* < 0.05) for TAN*S*.
- *M*: 0.388 (*p* < 0.05) for TAN*B*; 0.374 (*p* < 0.05) for TAN*V*; and 0.368 (*p* < 0.05) for TAN*S*.

**Figure 11:**
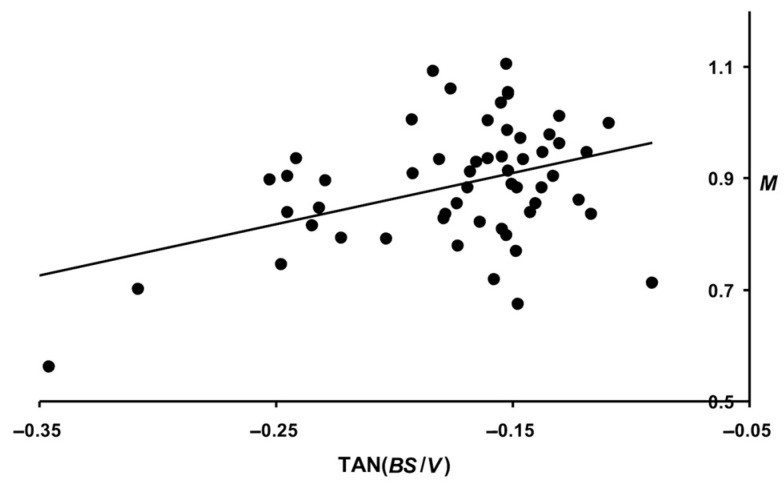
Correlation dependence of the total egg mass (*M*) for three months with the slope angles of the trend lines reflecting changes in the quail metabolic index, TAN(*B·S*/*V*).

A higher correlation was noted when analyzing the metabolic index that we proposed. In the event, the rate of its change (otherwise, the slope of the trend line) from 14 to 56 days of age demonstrated a relationship with *M* for three months of the productive period at the level of 0.443 (*p* < 0.05). A visualization of this relationship is presented in Figure 11.

The plotted dependencies in Figure 5, Figure 6, Figure 8, Figure 10 and Figure 11 show the general trends in the relationship between the parameters studied. Using them, performing the predictive calculations of quail productivity indicators is complicated by the insufficiently high accuracy of the obtained results. Nevertheless, such a calculation can have very relevant practical significance. In this regard, we decided to use the principle of synergy and combine the three key parameters of the bird (*B*, *V*, and *S*) into a single predictive calculation equation. As a result, the following multiparameter dependencies were obtained, providing the most accurate prediction outputs:

(10) $$N=412.515\frac{V^{0.041}}{B^{0.141}S^{0.227}},$$

with *R* = 0.725 (*p* < 0.05);

(11) $$W=9.673\frac{{(\mathrm{TAN}B)}^{0.03}\cdot {(\mathrm{TAN}V)}^{0.175}}{{(\mathrm{TAN}S)}^{0.04}},$$

with *R* = 0.524 (*p* < 0.05);

(12) $$M=0.727\frac{{(\mathrm{TAN}B)}^{0.183}\cdot {(\mathrm{TAN}S)}^{0.138}}{{(\mathrm{TAN}V)}^{0.138}},$$

with *R* = 0.448 (*p* < 0.05), where *N* is measured in eggs, *W* is in g, *M* is in kg, *B* is in g, *V* is in cm^3^, *S* is in cm^2^, and TAN*B*, TAN*V,* and TAN*S* are in radians.

An analysis of the resultant Equations (10)–(12) showed that their accuracy for the predictive calculation results is higher than that of a single-parameter prediction, even in the case of using the complex metabolic index (*B*·*S*/*V*). The only doubt regarding the prediction accuracy can be raised for Equation (12) that had the correlation coefficient of the calculation results with the true values below 0.5. In this respect, we made an attempt to improve the results by selecting the most adequate approximation equation to the data shown in Figure 11. As a result, a parabolic function was obtained, and the calculation of the values of which made it possible to increase the correlation coefficient (*R*) to 0.544 (*p* < 0.05):

(13) $$M=0.684-2.793\left(\mathrm{TAN}\frac{B\cdot S}{V}\right)-8.586{\left(\mathrm{TAN}\frac{B\cdot S}{V}\right)}^{2},$$

where *M* is measured in kg, and TAN(*B·S*/*V*) in radians.

Thus, Equations (10), (11), and (13) can be recommended for the practical predictive calculations of quail production indicators, i.e., *N*, average *W* of eggs laid, and *M*.

## 4. Conclusions

The metabolism level and its relationship to egg production in poultry has historically been understudied, perhaps undeservedly as it has a possible utility as a predictive factor. The metabolism level can be indirectly determined as the ratio of *S* to *V* of the bird. The method developed herein for determining these morphometric parameters in quail is based on the use of formulae for calculating ellipsoids, for which the bird’s *l* and *c* are measured.

The current study of the egg productivity in eight quail breeds confirmed the relationship of *N*, average *W,* and the resultant *M* value with the main body measurement parameters of the birds, i.e., their *B*, *V,* and *S*. Based on the mathematical analysis carried out in this study, a metabolic index was proposed, including the relationship between these three morphometric indicators (*B·S*/*V*).

It has been suggested that when predicting quail productivity indicators, i.e., *N*, average *W* of laid eggs, and *M*, it is advisable to use not only the values of the direct measurements and/or calculations of poultry morphometric parameters but also the dynamics of their changes. In addition to the single main parameters of quails, i.e., *B*, *V,* and *S* of the body, we noted a good analytical and predictive potential for including the trend of changes in these parameters during the period of 1–8 weeks of life, namely, the slope angles of their trend lines: TAN*B*, TAN*V*, and TAN*S*. The use of the trend of changes in the metabolic index TAN(*B*·*S*/*V*), especially when calculating the produced *M* value, was no less promising. Thus, we have developed a simple means of predicting egg productivity in quails, based on easily and quickly measurable morphometric parameters in laying hens that could be of great value to the poultry breeding industry.

## Figures and Tables

**Table 1:** Japanese quail breeds used in the study and their description.

Breed	Code	*n* ^1^	Origin	Performance ^2^	Refs
Pharaoh 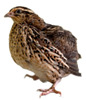	PHA	7	The USA; wild type color; a French fattening line that was imported and used in this study	*Meat type* *N* = 250 eggs; *W* = 13.8 g; *B_m_* = 200.2 g, *B_f_* = 260.5 g	[19,20,21,22,23,24]
Texas White (or Texas Pharaoh, White Pharaoh, Snowy) 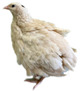	TEW	8	Texas, USA; from a cross between PHA and ENW	*Meat type* *N* = 243 eggs; *W* = 13.9 g; *B_m_* = 240.0 g, *B_f_* = 280.0 g	[22,23,24,25,26]
Estonian 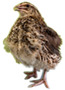	EST	9	Estonia, 1988; from a cross between JAP (a Moscow line), ENW and Pharaoh	*Dual purpose (or universal)* *N* = 263 eggs; *W* = 13.2 g; *B_m_* = 187.8 g, *B_f_* = 239.5 g	[21,22,23,27]
English White 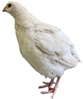	ENW	12	England; a mutant from JAP quails; brought from Hungary to the USSR in 1987	*Egg type* *N* = 261 eggs; *W* = 12.0 g; *B_m_* = 155.2 g, *B_f_* = 188.0 g	[19,21,22,23,28]
English Black 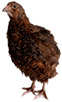	ENB	2	England; a mutant from JAP quails; brought from Hungary to the USSR in 1971	*Egg type* *N* = 261 eggs; *W* = 12.1 g; *B_m_* = 160.0 g, *B_f_* = 190.5 g	[21,22,23]
Tuxedo 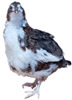	TUX	7	from a cross between ENW and ENB	*Egg type* *N* = 257 eggs; *W* = 12.0 g; *B_m_* = 148.2 g, *B_f_* = 180.5 g	[19,21,22,23]
Japanese 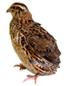	JAP	15	Japan; domesticated in Japan and China around 12th century or earlier; selected in the first half of the 20th century, imported to the USSR from Japan in the middle of the 20th century and/or from Yugoslavia in 1964	*Egg type* *N* = 288 eggs; *W* = 12.0 g; *B_m_* = 150.0 g, *B_f_* = 180.0 g	[19,21,22,23,27,29,30]
Manchurian Golden 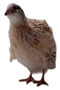	MAG	4	Marsh Farms, CA, USA, 1960s; selected by Albert Marsh in a flock of brown quails as a natural mutant	*Egg type* *N* = 267 eggs; *W* = 12.4 g; *B_m_* = 158.2 g, *B_f_* = 189.3 g	[19,21,22,23,31,32]

^1^ *n*, number of individuals. ^2^ *N*, egg production per year; *W*, egg weight at 10 weeks; body weight (*B*) at 6 weeks: *B_m_*, males, and *B_f_*, females. Breed data from Genofond LLC [21]. Image credit: authors’ own photographs.

**Table 2:** Body measurements of quails by days (1, 7, 14, 21, 28, 35, 42, 49, and 56) of their growth and their productivity indicators (mean value ± SD).

Parameters	Quail Breeds							
	TEW (a)	EST (b)	PHA (c)	MAG (d)	ENW (e)	JAP (f)	TUX (g)	ENB (h)
Body measurements								
Body weight in g (*B*)								
*B*_1_	9.6 ± 0.2 ^e,g^	9.1 ± 1.1	9.4 ± 0.3 ^e^	9.2 ± 0.6	8.3 ± 0.5 ^a,c,d^	9.0 ± 1.6	8.8 ± 0.7 ^a^	9.1 ± 0.7
*B*_7_	18.0 ± 5.1 ^e,f,g^	17.0 ± 2.3 ^e,f,g,h^	15.6 ± 6.8 ^f,g,h^	13.6 ± 4.2 ^f,h^	11.1 ± 4.3 ^a,b,f,h^	6.0 ± 1.3 ^a,b,c,d,e,g,h^	8.4 ± 1.5 ^a,b,c,d,e,f^	7.6 ± 0.4 ^a,b^
*B*_14_	61.4 ± 15.5 ^b,e,g,h^	42.2 ± 8.3 ^a,c,f,g^	62.2 ± 16.7 ^b,e,g,h^	47.8 ± 10.5 ^g^	44.4 ± 12.2 ^a,c,g^	51.7 ± 8.3 ^b,g,h^	27.6 ± 5.2 ^a,b,c,d,e,f,h^	37.0 ± 0.1 ^a,f,g^
*B*_21_	102.4 ± 6.1 ^b,e,f,g,h^	78.0 ± 14.1 ^a,c,f,g,h^	102.0 ± 22.2 ^b,f,g,h^	86.8 ± 15.3 ^f,g,h^	86.5 ± 14.5 ^a,f,g,h^	60.5 ± 3.5 ^a,b,c,d,e,g^	54.4 ± 6.4 ^a,b,c,d,e,h^	65.5 ± 3.5 ^a,e^
*B*_28_	183.3 ± 9.1 ^b,d,e,f,g,h^	134.1 ± 13.4 ^a,c,f,g,h^	165.4 ± 33.1 ^e,f,h^	146.0 ± 22.5 ^a,f,h^	119.3 ± 17.9 ^a,c,f,g^	94.6 ± 10.1 ^a,b,c,d,e,g^	124.9 ± 15.2 ^a,b,c,f,h^	94.0 ± 15.6 ^a,g^
*B*_35_	216.4 ± 8.0 ^b,d,e,f,g,h^	159.6 ± 10.8 ^a,c,e,f,g,h^	196.6 ± 28.9 ^b,e,f,g,h^	161.5 ± 39.3 ^a^	141.5 ± 18.6 ^a,b,c,f^	120.5 ± 13.4 ^a,b,c,e,h^	135.5 ± 15.4 ^a,b,c^	131.0 ± 0 ^a,b,c^
*B*_42_	261.8 ± 12.6 ^b,c,d,e,f,g,h^	175.1 ± 13.9 ^a,c,d,f,g,h^	221.1 ± 27.4 ^a,b,d,e,f,g,h^	191.7 ± 8.1 ^a,e,f,g,h^	159.6 ± 22.0 ^a,c,d,f,h^	133.0 ± 1.4 ^a,b,c,d,e,g^	146.1 ± 16.2 ^a,b,c,d^	137.0 ± 8.5 ^a,b,c,d^
*B*_49_	277.0 ± 18.4 ^b,c,d,e,f,g,h^	193.7 ± 15.7 ^a,c,e,f,g,h^	243.6 ± 32.0 ^a,b,d,e,f,g,h^	198.3 ± 7.6 ^a,c,e,f,g,h^	167.7 ± 16.1 ^a,b,c,d,f,h^	135.5 ± 3.5 ^a,b,c,d,e,g^	164.0 ± 13.6 ^a,b,c,d,f^	144.0 ± 9.9 ^a,b,c,d^
*B*_56_	312.9 ± 24.7 ^b,c,d,e,f,g,h^	205.4 ± 13.5 ^a,c,e,f,g,h^	253.4 ± 34.2 ^a,b,d,e,f,g,h^	207.7 ± 11.8 ^a,c,e,f,g,h^	181.0 ± 21.1 ^a,b,c,d,f,h^	160.1 ± 16.8 ^a,b,c,d,e^	164.1 ± 21.9 ^a,b,c,d^	154.5 ± 2.1 ^a,b,c,d^
Body length in cm (*l*)								
*l*_14_	6.0 ± 0.8 ^c^	5.8 ± 0.6 ^c^	7.0 ± 0.6 ^a,b,e,f,g,h^	6.7 ± 0.8	6.1 ± 0.7 ^c^	6.0 ± 0.4 ^c^	5.8 ± 0.7 ^c^	6.0 ± 0.4
*l*_28_	8.9 ± 0.8 ^e,f,g,h^	8.1 ± 0.7 ^c^	8.2 ± 0.4 ^e,f,g,h^	8.4 ± 0.9 ^g^	7.2 ± 0.7 ^a,b,c,d^	7.5 ± 0.6 ^a,c,g^	6.7 ± 0.4 ^a,b,c,d,e,f,h^	7.4 ± 0.1 ^a,c^
*l*_42_	9.5 ± 0.8 ^e,f,g,h^	8.9 ± 0.4 ^d,e,f^	9.5 ± 0.8 ^e,f,g,h^	9.3 ± 0.3 ^e,f^	8.5 ± 0.4 ^a,b,c,d^	8.1 ± 1.1 ^a,b,c,d,e^	8.7 ± 0.6 ^a,c,d^	8.4 ± 0.5
*l*_56_	10.3 ± 0.5 ^b,d,e,f,h^	9.6 ± 0.4 ^a,e,g,h^	10.3 ± 0.6 ^b,d,e,f,h^	10.0 ± 0.5 ^h^	8.9 ± 0.4 ^a,b,c,g^	9.1 ± 1.2 ^a,c,g^	10.5 ± 0.6 ^a,b,d,e,f,h^	8.8 ± 0.0 ^a,b,c,g^
Body circumference in cm (*c*)								
*c*_14_	9.2 ± 0.6 ^b,f,g,h^	8.4 ± 0.6 ^a,c,f,g,h^	9.6 ± 1.1 ^b,f,g,h^	8.5 ± 0.9 ^g^	8.4 ± 1.2 ^c,g,h^	7.7 ± 0.2 ^a,b,c,e,g,h^	6.5 ± 0.5 ^a,b,c,d,e,f,h^	7.2 ± 0.1 ^b,c^
*c*_28_	13.5 ± 1.3 ^b,e,f,g,h^	11.3 ± 0.7 ^a,c,g,h^	12.6 ± 1.0 ^b,e,f,g,h^	12.0 ± 1.2 ^g^	11.3 ± 0.8 ^a,c,g,h^	11.1 ± 0.6 ^a,c,g,h^	9.9 ± 0.8 ^a,b,c,d,e,f^	10.6 ± 0.2 ^a,c^
*c*_42_	17.3 ± 0.2 ^b,d,e,f,g,h^	15.2 ± 0.6 ^a,c,f,g,h^	16.5 ± 1.0 ^b,e,f,g,h^	15.4 ± 0.2 ^a,e,f,g,h^	14.4 ± 0.8 ^a,b,c,d,f,g,h^	13.5 ± 0.2 ^a,b,c,d,e,g^	13.8 ± 0.4 ^a,b,c,d,f^	13.6 ± 0.2 ^a,b,c,d^
*c*_56_	18.3 ± 0.6 ^b,d,e,f,g,h^	16.0 ± 0.7 ^a,c,e,f,g,h^	17.3 ± 1.0 ^b,d,e,f,g,h^	16.4 ± 0.3 ^a,e,f,g,h^	15.2 ± 0.7 ^a,b,c,d,f,h^	14.2 ± 0.6 ^a,b,c,d,e^	14.7 ± 0.6 ^a,b,c,d^	14.2 ± 0.4 ^a,b,c,d^
Body volume in cm^3^ (*V*)								
*V*_14_	27.3 ± 7.1 ^f,g,h^	22.1 ± 4.8 ^c,g,h^	34.9 ± 9.1 ^b,f,g,h^	26.3 ± 8.3 ^g^	23.6 ± 7.6 ^c,g,h^	18.7 ± 2.4 ^a,c,e,g,h^	13.3 ± 3.2 ^a,b,c,d,e,f,h^	16.3 ± 0.3 ^a,c^
*V*_28_	87.9 ± 23.7 ^b,e,f,g,h^	54.9 ± 8.8 ^c,g,h^	70.1 ± 11.6 ^b,e,f,g,h^	65.0 ± 16.5 ^g^	49.5 ± 9.8 ^a,c,g^	49.7 ± 8.1 ^a,c,g,h^	35.5 ± 7.2 ^a,b,c,d,e,f,h^	43.7 ± 2.6 ^a,c^
*V*_42_	151.9 ± 13.8 ^b,d,e,f,g,h^	111.2 ± 14.6 ^a,c,d,e,f,g,h^	140.0 ± 26.4 ^b,e,f,g,h^	117.6 ± 6.3 ^a,e,f,g,h^	93.6 ± 11.7 ^a,b,c,d,f,h^	77.4± 12.6 ^a,b,c,d,e,g^	88.3 ± 9.6 ^a,b,c,d^	81.3 ± 2.3 ^a,b,c,d^
*V*_56_	183.7 ± 19.0 ^b,d,e,f,g,h^	128.5 ± 10.2 ^a,c,e,f,h^	161.3 ± 20.5 ^b,d,e,f,g,h^	142.5 ± 3.5 ^a,e,f,g,h^	110.1 ± 9.9 ^a,b,c,d,f,h^	96.9 ± 14.1 ^a,b,c,d,e,g^	120.7 ± 16.3 ^a,c,f,h^	94.2 ± 5.6 ^a,b,c,d^
Body surface area in cm^2^ (*S*)								
*S*_14_	47.2 ± 8.8 ^f,g,h^	41.5 ± 6.4 ^c,g,h^	56.7 ± 10.1 ^b,f,g,h^	47.7 ± 10.6 ^g^	43.5 ± 10.0 ^c,g,h^	38.3 ± 3.6 ^a,c,g,h^	31.2 ± 5.4 ^a,b,c,d,e,f^	35.5 ± 1.2 ^c^
*S*_28_	102.9 ± 18.1 ^b,e,f,g,h^	76.9 ± 8.4 ^c,g,h^	88.8 ± 9.1 ^b,e,f,g,h^	85.3 ± 15.0 ^g^	70.0 ± 9.8 ^a,c,g^	71.1 ± 8.1 ^a,c,g^	56.8 ± 7.3 ^a,b,c,d,e,f,h^	65.9 ± 2.6 ^a,c^
*S*_42_	144.4 ± 10.3 ^b,d,e,f,g,h^	118.9 ± 11.0 ^a,c,d,e,f,g,h^	137.9± 18.3 ^b,e,f,g,h^	123.7 ± 4.8 ^a,e,f,g,h^	105.7 ± 8.2 ^a,b,c,d,f,h^	93.5 ± 12.1 ^a,b,c,d,e,g^	102.9 ± 8.3 ^a,b,c,d^	97.1 ± 3.2 ^a,b,c,d^
*S*_56_	164.4 ± 11.5 ^b,d,e,f,g,h^	130.7 ± 6.0 ^a,c,e,f,h^	151.5 ± 12.6 ^b,d,e,f,g,h^	141.1 ± 3.4 ^a,e,f,g,h^	117.7 ± 6.7 ^a,b,c,d,h^	109.9 ± 13.0 ^a,b,c,d,g^	130.1 ± 12.2 ^a,c,e,f,h^	107.2 ± 3.7 ^a,b,c,d,g^
Metabolic level (*S*/*V*)								
*S*_14_/*V*_14_	1.76 ± 0.13 ^f,g,h^	1.91 ± 0.14 ^a,c,f,g,h^	1.67 ± 0.21 ^b,f,g,h^	1.87 ± 0.22 ^g^	1.94 ± 0.35 ^g,h^	2.06 ± 0.07 ^a,b,c,g,h^	2.39 ± 0.19 ^a,b,c,d,e,f,h^	2.17 ± 0.03 ^a,b,f^
*S*_28_/*V*_28_	1.19 ± 0.11 ^b,e,f,g,h^	1.41 ± 0.09 ^a,c,f,g,h^	1.28 ± 0.09 ^b,e,f,g,h^	1.34 ± 0.13 ^g^	1.43 ± 0.11 ^a,c,g^	1.44 ± 0.08 ^a,c,g,h^	1.62 ± 0.12 ^a,b,c,d,e,f^	1.51 ± 0.03 ^a,c^
*S*_42_/*V*_42_	0.95 ± 0.02 ^b,d,e,f,g,h^	1.07 ± 0.04 ^a,c,e,f,g,h^	0.99 ± 0.06 ^b,e,f,g,h^	1.05 ± 0.02 ^a,e,f,g,h^	1.14 ± 0.05 ^a,b,c,d,f,h^	1.21 ± 0.04 ^a,b,c,d,e,g^	1.17 ± 0.03 ^a,b,c,d,f^	1.19 ± 0.01 ^a,b,c,d^
*S*_56_/*V*_56_	0.90 ± 0.03 ^b,c,d,e,f,g,h^	1.02 ± 0.04 ^a,c,e,f,g,h^	0.94 ± 0.05 ^b,d,e,f,g,h^	0.99 ± 0.01 ^a,e,f,g,h^	1.07 ± 0.04 ^a,b,c,d,f,h^	1.14 ± 0.05 ^a,b,c,d,e,g^	1.08 ± 0.05 ^a,b,c,d,f^	1.14 ± 0.03 ^a,b,c,d^
Quail productivity for 3 months: egg production (*N*); average egg weight in g (*W*); and total egg mass in kg (*M*)								
*N*	69.5 ± 7.1 ^b,d,e,f,g,h^	78.9 ± 5.6 ^a,c,f^	73.0 ± 4.1 ^b,d,e,f,g,h^	80.7 ± 3.5 ^a,c^	81.7 ± 3.8 ^a,c^	83.9 ± 3.2 ^a,b,c^	83.0 ± 4.8 ^a,c^	84.0 ± 2.8 ^a,c^
*W*	11.2 ± 1.2	11.5 ± 1.4 ^f^	12.5 ± 0.8 ^e,f,h^	12.3 ± 0.6 ^e,f,h^	11.0 ± 0.8 ^c,d,f^	10.1 ± 0.8 ^a,b,c,d,e,g^	11.6 ± 0.9 ^f^	10.9 ± 0.5
*M*	0.78 ± 0.12 ^c,d,e,g^	0.91 ± 0.16	0.91 ± 0.08 ^a^	0.99 ± 0.05 ^a,f^	0.90 ± 0.07 ^a,d^	0.85 ± 0.08 ^d,g^	0.97 ± 0.10 ^a,f^	0.91 ± 0.07

Breeds: TEW, Texas White; EST, Estonian; PHA, Pharaoh; MAG, Manchurian Golden; ENW, English White; JAP, Japanese; TUX, Tuxedo; and ENB, English Black. ^a,b,c,d,e,f,g,h^ *p* < 0.05 for the respective breed; the absence of a corresponding superscript indicates that the values for any two breeds are insignificant. More specifically, each breed is assigned a letter symbol, i.e., ^a,b,c,d,e,f,g,h^, respectively. The presence of a specific index (indices) conforms to significant differences with a given breed at *p* < 0.05. The absence of a certain index indicates insignificant differences with a given breed.

## Data Availability

The data presented in this study are openly available in FigShare at 10.6084/m9.figshare.24994760, reference number 24994760.
